# Interplay between male quality and male-female compatibility across episodes of sexual selection

**DOI:** 10.1126/sciadv.adf5559

**Published:** 2023-09-29

**Authors:** Hayat Mahdjoub, Rassim Khelifa, Jeannine Roy, Sonja H. Sbilordo, Valérian Zeender, Jhoniel Perdigón Ferreira, Natalia Gourgoulianni, Stefan Lüpold

**Affiliations:** ^1^ Department of Evolutionary Biology and Environmental Studies, University of Zurich, Winterthurerstrasse 190, 8057 Zurich, Switzerland.; ^2^ Biology Department, Concordia University, 7141 Sherbrooke St. W., Montreal QC H4B 1R6, Canada.

## Abstract

The processes underlying mate choice profoundly influence the dynamics of sexual selection and the evolution of male sexual traits. Consistent preference for certain phenotypes may erode genetic variation in populations through directional selection, whereas divergent preferences (e.g., genetically compatible mates) provide one mechanism to maintain such variation. However, the relative contributions of these processes across episodes of selection remain unknown. Using *Drosophila melanogaster*, we followed the fate of male genotypes, previously scored for their overall reproductive value and their compatibility with different female genotypes, across pre- and postmating episodes of selection. When pairs of competitor males differed in their intrinsic quality and their compatibility with the female, both factors influenced outcomes from mating success to paternity but to a varying degree between stages. These results add further dimensions to our understanding of how the interactions between genotypes and forms of selection shape reproductive outcomes and ultimately reproductive trait evolution.

## Teaser

In *D. melanogaster*, male quality and male-female compatibility differentially affect sexual selection across reproductive stages.

## INTRODUCTION

A central debate in evolutionary and behavioral ecology revolves around why and how mate choice has evolved and how it benefits choosing individuals (*1*–*4*). Predictions about female mate choice are relatively straightforward in resource-based mating systems in which males provide material benefits to females or offspring, such as food, parental care, or protection against predators (*1*). In many species, however, males provide no resources, and females are predicted to benefit exclusively from genetic contributions of their partners that confer higher fitness on their offspring (*5*). Females can coincide in their preference for certain males [e.g., with the most elaborate traits linked to male vigor; (*6*, *7*)], or they can differ in their choice, with genetic benefits derived from favorable interactions between the parental genomes [or a reduction of incompatible allele combinations (*8*–*12*)]. Unlike the shared female preferences for intrinsically superior males that are thought to deplete genetic variation through persistent directional selection [i.e., “lek paradox;” (*7*, *13*, *14*)], preferences based on genomic compatibility provide one important explanation for the maintenance of genetic variation (*14*, *15*). Reinforcement of preferences against incompatible combinations further has the potential to cause reproductive isolation and so facilitate speciation (*16*, *17*).

Choosing mates before copulation is a critical first filter toward enhancing indirect benefits. However, choice is contingent on the temporal mate availability, and information on potential genetic benefits is likely to be both indirect (e.g., via trait expression) and incomplete in that genotype-environment interactions resulting from environmental heterogeneity can disrupt the reliability of signals of genetic quality (*18*, *19*). By mating with multiple males, females can gain additional opportunities to bias reproductive outcomes among those males that did mate (*20*–*22*). Simply facilitating competition among sperm from different mates can bias fertilization success toward functionally superior sperm or toward sperm carrying haplotypes that are more compatible with those of the female (*23*–*25*). Females, however, might also play a more active role by differentially storing sperm and using them for fertilization (*20*, *22*), for example, based on molecular interactions between their reproductive tract secretions and ejaculates or between sperm and eggs during the fertilization process (*26*, *27*). Through either mechanism, females can accrue indirect benefits if the fertilizing sperm carry fitness-enhancing alleles—directly or in combination with the egg haplotype—which are then inherited by the offspring (*23*–*25*). Postmating biases thus have the potential to reinforce premating decisions by enhancing the fertilization success of preferred males [e.g., (*28*)]. However, postmating processes can also modify initial mating decisions. For example, if genomic complementarity is difficult to detect before copulation, mating with multiple males that pass a female’s quality threshold would permit secondary selection against less compatible genotypes among those mates via postmating processes. A disconnect between mate choice and gametic compatibility has been proposed as one cause of infertility in humans (*29*).

Despite a wealth of studies on the causes and consequences of mate choice [reviewed in (*3*)], there remains an ongoing debate about the relative importance of additive mate effects (thought to capture some degree of intrinsic male “quality” or reproductive value] and male-female interacting effects (often considered “genetic compatibility,” besides dominance and epistasis) (*4*, *8*, *9*, *30*). Many studies use breeding designs that are powerful for partitioning observed phenotypic variation into these additive and nonadditive genetic effects at the population level (*9*, *31*). However, the commonly used noncompetitive mating trials in these studies do not inform about how females bias reproductive outcomes when exposed to males that vary in both their intrinsic quality and their genetic compatibility with the choosing female.

Furthermore, the relative importance of intrinsic male quality and genetic compatibility, as well as of mate choice and mate competition, is likely to differ between the pre- and postmating episodes of selection. For example, compared to the diverse condition-dependent indicator traits thought to display additive benefits to females (*1*, *3*), genetic compatibility is more difficult for males to advertise because of its inherent nontransitivity. Signaling genetic compatibility should be largely restricted to chemical communication, which can occur between single molecules and receptors expressed by single genes, unlike other signaling modalities that necessarily involve complex, polygenic stimulus traits or sensory organs (*32*, *33*). Variation in male quality is thus likely to play a far more important role than genetic compatibility in premating female choice. By contrast, there is much greater potential for assessment of genetic compatibility during postmating stages through the numerous physical and biochemical interactions between ejaculates and the female reproductive tract or ova (*27*, *29*, *34*). Even if a female does not actively discriminate among rival sperm, the selective environment of her reproductive tract (e.g., morphology, physiology, and biochemistry) might be more favorable to some sperm than others, thereby indirectly enhancing their fertilization success in response to some female attributes (*35*). Disentangling the contributions of male quality and genetic compatibility to female biases thus requires following the fate of male genotypes of known relative quality and compatibility throughout the different reproductive stages under competitive conditions. The contributions of pre- and postmating processes of selection to paternity shares between males varying in quality and compatibility, however, are not usually examined simultaneously. Rather, studies typically aim to distinguish between additive and nonadditive effects at either the mate or gamete level (*10*, *30*) or to partition the (typically male-specific) variance in total reproductive success between episodes of selection without quantifying male-female genotypic interactions (*36*, *37*). Such separation hampers direct comparisons of different genetic contributions across reproductive stages and, ultimately, our understanding of the mechanisms underlying female choice (*9*, *11*).

Here, we used a multistage experimental approach to assess the relative importance of male quality and male-female compatibility across the pre- and postmating episodes of selection in *Drosophila melanogaster*. In this species, males provide no material benefits to females. Rather, female interactions with males and, particularly mating, can be detrimental (*38*–*42*). Yet, males have been reported to benefit females indirectly by enhancing different offspring fitness components (*43*–*46*) [but see (*47*, *48*)], although such paternal effects are not necessarily genetic (*49*–*51*) and may not offset the costs of mating (*52*). Female mate choice is transitive (i.e., consistent male ranking) (*48*), influenced by male courtship and pheromones (*53*, *54*). Female cooperation is essential for copulation to occur (*55*), meaning that male coercion is extremely rare (*56*, *57*). After copulation, females can bias competitive fertilization success among males either actively through the timing and extent of sperm ejection (*58*) or passively through variation in reproductive tract morphology (*59*). These biases contribute to three-way interactions in reproductive outcomes between genetically varying females and pairs of competitor males, suggesting a high degree of nontransitivity at the postmating stage that could override initial mating decisions (*37*, *60*).

In our study, we first conducted a range of fitness assays for each possible pairwise cross between 20 male and 10 female genotypes (at *N* = 8 to 15 replicates per combination; fig. S1, A to C), with each genotype derived from a pairwise cross between two unique isogenic lines (i.e., heterozygous but quasi-clonal). These assays generated gradients in the reproductive performance of male-female genotypic combinations and of male genotypes across all female genotypes. For simplicity, we will henceforth refer to these effects as male-female compatibility and male quality, respectively. It is important to note, however, that high-quality scores do not exclusively reflect intrinsic male quality but could also include components of congruent female preference (e.g., male attractiveness) or particularly widespread male compatibility with female genotypes. Because our main goal was not to distinguish between different types of indirect benefits but to quantify the relative importance of male quality and male-female compatibility in reproductive biases, we considered overall variation among male genotypes to reflect differences in male quality or reproductive value. Next, we staged competitive matings between males that differed in their overall quality scores and their nonadditive scores with the corresponding focal female and then followed their biases in reproductive outcomes across the pre- and postmating episodes of selection (fig. S1, D to F). Green (GFP) or red fluorescent protein (RFP) expressed in the sperm heads allowed us to track the spatiotemporal distribution of sperm across the different postmating processes (*35*, *58*, *61*, *62*). We predicted that male genotypic variation should, overall, have a stronger effect on reproductive outcomes than male-female interactions (*63*) but that the latter would primarily come into play after copulation because of greater potential for intricate molecular interactions between genotypes. To this end, we estimated premating female choice by relative mating success (MS) between competing male genotypes (fig. S1D) and postmating female biases as the time between mating and female sperm ejection (fig. S1E) (*58*), given that competitive fertilization success is typically proportional to relative sperm numbers in storage (*35*, *58*, *61*, *62*). We also estimated the proportion of second-male paternity (P_2_) by sequentially mating females with two males (one GFP and one RFP) that varied in their difference in intrinsic quality and male-female compatibility across mating combinations (fig. S1F).

## RESULTS

**Fig. 1.**
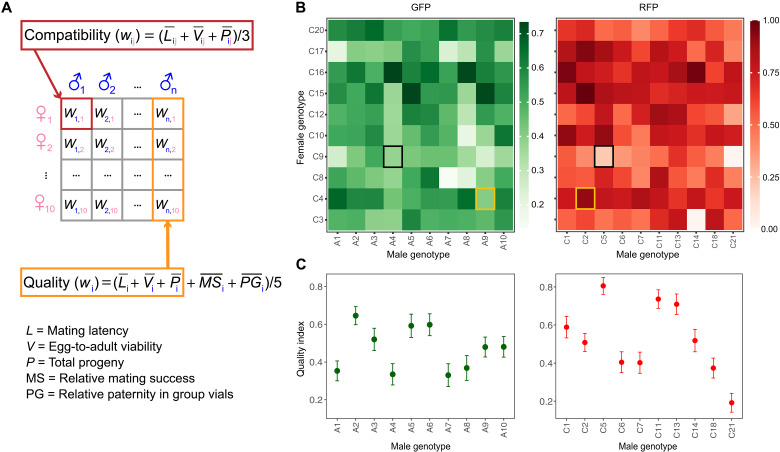
Indices of male-female compatibility and male quality in our experimental heterozygous genotypes of *Drosophila melanogaster*. (**A**) Depiction of how the compatibility (*w*_ij_) and quality indices (*w*_i_) were calculated on the basis of the three or five fitness variables, respectively. Each variable was first range-standardized before averaging. The red box shows the calculation of male-female compatibility, the orange box that of male quality (i.e., MS and PG added to the average over the 10 female genotypes for all other variables). (**B**) Male-female compatibility index for each male-female pairwise combination of 10 RFP female genotypes with either 10 GFP (left) or 10 RFP male genotypes (right). In all cases, heterozygous genotypes were generated by crossing pairs of independent isogenic GFP or RFP lines, respectively. (**C**) Quality index for each male genotype based on the calculation in (A), with bootstrapped 95% confidence intervals. After selecting male genotypes with varying differences in quality and compatibility, we performed a precopulatory and postcopulatory competitive experiment where an RFP female was paired simultaneously or sequentially with a GFP and an RFP male (fig. S1). Two hypothetical combinations are indicated by colored squares in (B): female (genotype C4) combined with two males of similar quality but differential male-female compatibility scores (yellow) or female (C9) combined with two males of similar compatibility but differential quality scores (black).

We performed all our experiments with *D. melanogaster* (wild-type strain LH_m_) that were genetically transformed so that sperm heads express either GFP or RFP (*61*). We first determined the fitness of male genotypes and male-female genotypic combinations by crossing each of 10 GFP and 10 RFP male genotypes with 10 RFP female genotypes in all pairwise combinations, replicating all 200 male-female combinations five times in each of three time-separated blocks (fig. S1A). The averaged standardized mean values of mating latency [“*L*”: widely used proxy of male “attractiveness” (*57*, *64*); here, additive inverse so that higher scores reflected faster mating initiation], egg-to-adult viability (“*V*”), and the total progeny number (“*P*”) provided a combination-specific fitness value (i.e., compatibility score; Fig. 1, A and B, and figs. S1 to S3). Using a bootstrapping approach and repeated-measures correlations (*65*), egg-to-adult viability covaried positively with the total progeny number [*r* = 0.37 (95% confidence interval: 0.25 to 0.48)], whereas the other two pairs of traits were not correlated [*L* − *V*: *r* = −0.06 (−0.17 to 0.06); *L* − *P*: *r* = 0.04 (−0.07 to 0.14)].

To obtain overall scores of intrinsic male quality (i.e., reproductive value), we averaged the standardized values of the above three variables for each male genotype across all 10 female genotypes and combined these with two standardized, genotype-specific scores from competitive assays (separately for each male color): David’s scores [i.e., “dominance” ranks; (*66*, *67*)] derived from competitive mating trials (“MS”: two males, one virgin female] between all male-male combinations, and the proportion of focal-male paternity in group vials (“PG”: one focal male and two standard competitors of the opposite fluorescent tag allowed to interact freely with three standard females for 6 days) [Fig. 1 (A and C) and figs. S1 (B and C), S4, and S5]. Bootstrapped Spearman’s rank correlations across the 20 male genotypes revealed that (inverse) mating latency (*L*) covaried positively with total progeny production [*P*; ρ = 0.69 (0.54 to 0.81)] and competitive paternity (PG) [ρ = 0.30 (0.01 to 0.57)] and tended to do so with competitive MS [ρ = 0.16 (−0.02 to 0.35); table S1]. Note that high scores by certain male genotypes in these assays can again include contributions of higher competitiveness by and consistent female preference for these males.

On the basis of the male- and combination-specific fitness indices (Fig. 1, A to C), we then staged competitive matings between GFP and RFP males that contrasted in their intrinsic fitness or their relative fitness with the given female genotype. We quantified the relative contributions of these qualities (based on five traits) to MS in simultaneous competitive mating trials as above and to postmating processes and fitness outcomes in sequential mating trials (see Materials and Methods and fig. S1, D to F).

### Competitive MS

**Fig. 2.**
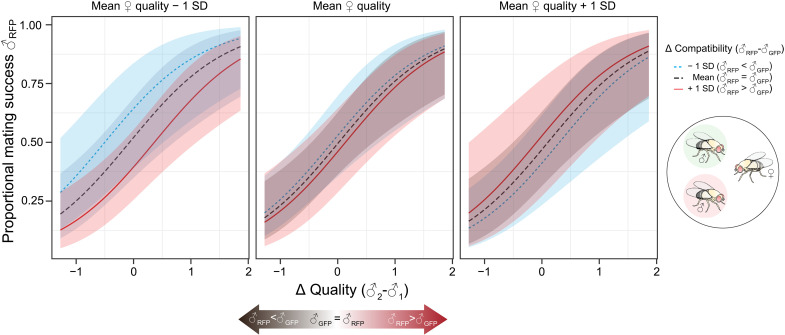
Competitive MS between males of varying quality and compatibility with the female. In each trial, an RFP and a GFP male that differed primarily in their quality or in their compatibility with the given female were competitively mated. The relative MS (RFP as the focal male) covaried with the difference (Δ) in male quality [♂_RFP −_ ♂_GFP_] and with an interaction between the difference in male-female compatibility and female quality. All predictor variables were standardized to ~*N*(0,1). To illustrate the effect of female quality and male-female compatibility, three levels are shown for each variable (mean, mean – 1 SD, and mean + 1 SD). The fitted values of relative MS are predicted from a generalized linear mixed-effects model.

To quantify the relative contributions of intrinsic male quality and male-female compatibility to MS, we set up mating trios of one female with one GFP and one RFP male that differed [♂_RFP_-♂_GFP_] to a varying degree in their intrinsic quality and their corresponding compatibility with the female genotype. On average, the male to mate with the female was the one with a higher quality score [generalized linear mixed model with binomial error distribution and log link, henceforth shortened to “binomial GLMM”: *N* = 167, β (± SE) = 1.18 ± 0.30, log-likelihood ratio (LLR) = 13.85, *P* = 0.0002; Fig. 2 and table S2]. In addition, female quality (based on their mean offspring production and viability across all 20 male genotypes) and the difference in male-female compatibility between competitors showed a weak interactive effect on MS (β = 0.52 ± 0.26, LLR = 3.94, *P* = 0.047).

### Sperm ejection latency

**Fig. 3.**
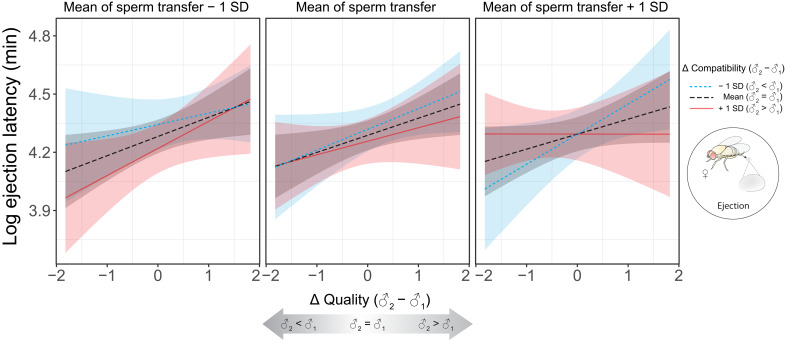
The effects of the difference (Δ) in male quality scores, difference (Δ) in male-female compatibility scores, and ejaculate size on ejection latency (log-transformed) in *D. melanogaster*. All predictor variables were standardized to ~*N*(0,1). Three levels of Δ compatibility and ejaculate size were used to illustrate their effect on the response variable, namely, mean, mean – 1 SD, and mean + 1 SD. The fitted values of relative MS are predicted from a linear mixed-effects model.

To study postmating processes and fitness outcomes, we sequentially mated each focal female to a GFP and RFP male (in reciprocal mating orders). After the second mating, we measured the time until the female ejected a mass containing displaced first-male and excess second-male sperm [ejection latency; (*58*, *61*, *68*)], which had previously been found to correlate positively with the proportion of second-male sperm among all sperm remaining in storage [i.e., S_2_; (*58*)]. In a linear mixed-effects model on 227 observations, the time to female sperm ejection was primarily explained by a three-way interaction between the number of second-male sperm transferred and the between-male differences (♂_2_ − ♂_1_) in both male quality and the compatibility with the female genotype (β = −0.11 ± 0.04, LLR = 2.71, *P* = 0.007; Fig. 3 and table S3). Specifically, females generally delayed sperm ejection if the second male was of higher quality than the first. With increasing second-male ejaculate size, this trend gradually shifted to being more pronounced if the second male was relatively less compatible with the female than the first.

### Second-male sperm proportion in the reproductive tract (S_2_)

**Fig. 4.**
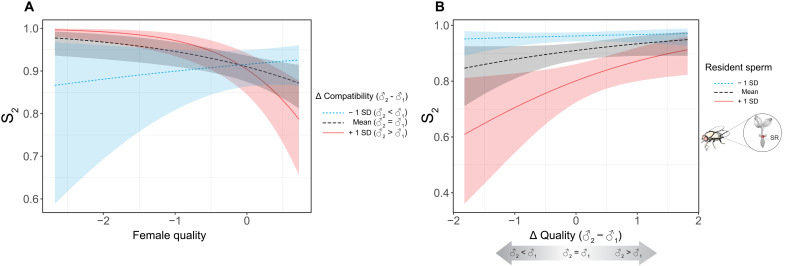
Male and female effects on relative sperm storage in *D. melanogaster*. Proportion of second-male sperm residing in the female seminal receptacle after sperm ejection in response to (**A**) an interaction between female quality and differential male-female compatibility and to (**B**) an interaction between the difference in male genotypic quality and the number of resident sperm in storage at remating. All predictor variables were standardized to ~*N*(0,1). Three color levels illustrate the effects on the response variable, namely, mean, mean – 1 SD, and mean + 1 SD. The fitted values were predicted from a generalized linear mixed-effects model.

For the same flies as for sperm ejection latency, we next determined potential biases in sperm storage, expressed as the proportion of the second male’s sperm among those remaining in the female reproductive tract (S_2_) after sperm ejection (*69*). Here, we focused particularly on the proportional representation of sperm within the seminal receptacle, which constitutes the population of sperm competing for fertilization and has been shown to directly predict paternity outcomes (*58*, *62*, *69*). In a binomial GLMM (*N* = 241 across the same genotypic combinations as above), S_2_ increased with second-male ejaculate size (β = 1.35 ± 0.20, LLR = 42.68, *P* < 0.001) but decreased with the number of first-male resident sperm (β = −1.72 ± 0.22, LLR = 54.84, *P* < 0.001). The difference in male compatibility had an interactive effect with female quality (β = −0.49 ± 0.22, LLR = 5.49, *P* = 0.019), indicating that females of below-average quality biased sperm storage more strongly toward the second male than higher-quality females (also see corresponding main effect: β = −0.51 ± 0.21, LLR = 4.94, *P* = 0.026), particularly if this male was relatively more compatible than his competitor (Fig. 4A and table S4). A trend for an interactive effect was also found between the difference in male quality and first-male resident sperm numbers (β = 0.34 ± 0.20, LLR = 3.23, *P* = 0.072). If true, this would suggest that when only few first-male sperm were in storage (i.e., strong numerical advantage for second male), the second male secured high S_2_ regardless of quality differences. However, when many first-male sperm still resided in storage (i.e., more intense and numerically less biased sperm competition), S_2_ became contingent on the difference in quality scores between males (Fig. 4B and table S4).

While this variation in sperm storage could be the result of female biases, it could also be argued that males differentially invested in ejaculate composition. We did not quantify variation in seminal fluid proteins, but based on total sperm numbers transferred by second males, any differences were not explained by the quality scores of either the male or female genotypes nor the compatibility between them (all LLR ≤ 0.17, *P* ≥ 0.681). Only GFP males tended to transfer slightly more sperm on average compared to RFP males (β = 135.77 ± 69.66, LLR = 2.91, *P* = 0.088; table S5).

### Second-male paternity (P_2_)

**Fig. 5.**
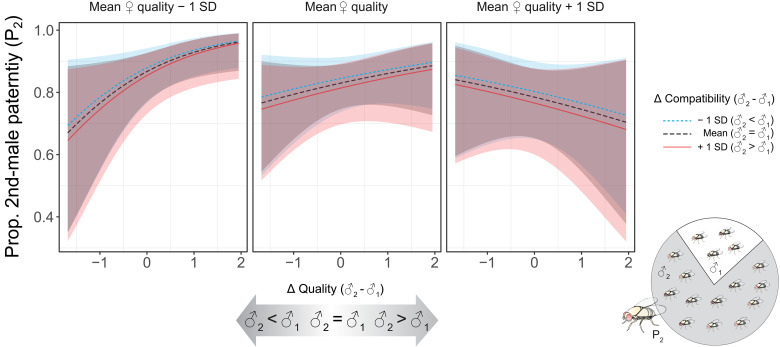
Male and female effects on the proportional paternity share of the second male (P_2_) in *D. melanogaster*. The contributing effects were the difference (Δ) in male quality and an interaction between the difference in male-female compatibility and female quality. All predictor variables were standardized to ~*N*(0,1). Three levels of Δ compatibility and female quality were used to illustrate their effects on the response variable, namely, mean, mean – 1 SD, and mean + 1 SD. The fitted values were predicted from a generalized linear mixed-effects model.

We quantified the fitness outcome of females sequentially mating with two males that varied in their difference in intrinsic quality and male-female compatibility. We estimated the proportion of P_2_ by allowing each female to lay eggs for 3 days and then counting her number of GFP and RFP progeny. Across all 214 females that remated and produced offspring, a binomial GLMM revealed a significant interaction between female quality and the difference in male quality (♂_2_ − ♂_1_) on P_2_ (β = −0.85 ± 0.26, LLR = 10.15, *P* = 0.001) but no effect of the difference in male-female compatibility (β = 0.16 ± 0.28, LLR = 1.22, *P* = 0.270). However, *N* = 44 of these females produced no offspring sired by the second male, thus leaving their remating status ambiguous. A more conservative estimate on the *N* = 170 females with confirmed mixed paternity indicated a consistent, albeit slightly weaker, interaction between female quality and differential male quality (β = −0.52 ± 0.22, LLR = 5.19, *P* = 0.023; table S6). Hence, females with below-average breeding value increasingly biased paternity toward the second male if he was of higher quality, whereas P_2_ was largely independent of male quality among higher-quality females (Fig. 5). Although the remating interval typically has a strong effect on P_2_ due to first-male sperm use (*35*, *58*, *61*, *62*) (also see above for S_2_), this variable dropped out during model simplification in both analyses (LLR ≤ 0.68, *P* ≥ 0.408), possibly because most females remated within 24 hours of one another.

Last, we compared the rate of offspring production before and after remating, expressed as the proportion of a given female’s total offspring produced that both males combined sired after remating as opposed to the offspring produced with the first male only before remating. This analysis essentially estimated whether the female’s offspring production rate was up-regulated after remating with a male of higher quality or compatibility relative to her first mate (using offspring production with the first male before remating as the baseline). A binomial GLMM on the same 170 observations and controlling for the remating interval (i.e., the duration of the first oviposition window; β = −0.74 ± 0.23, LLR = 10.41, *P* = 0.001) revealed that the daily offspring production increased significantly after remating (i.e., second oviposition window) if the second male was of higher quality than the first (β = 0.59 ± 0.15, LLR = 12.68, *P* < 0.001; fig. S6 and table S7). The difference in compatibility with the female had no statistically significant effect (β = 0.13 ± 0.12, LLR = 1.21, *P* = 0.27).

## DISCUSSION

We studied how male intrinsic quality and male-female compatibility influence different stages of pre- and postmating sexual selection in *D. melanogaster*, a species with no material benefits to females. We manipulated these two factors in a two-way factorial design and measured their effects on male MS and paternity outcomes under competitive conditions. We found that both factors influenced reproductive biases across episodes of selection, but their relative importance varied depending on the reproductive stage and female quality. Our results suggest that female *D. melanogaster* may use different criteria to bias reproductive outcomes across stages of the reproductive process, possibly in a context-dependent manner, and that both additive and nonadditive effects contribute to sexual selection.

MS, resulting from both premating male-male competition and female choice, was primarily biased toward the male with a relatively higher quality index than his competitor. Because our male quality index was based on mating latency, MS and sperm competitiveness, higher-quality males may, on average, have been of higher competitive vigor or more effective in inducing the female to mate (*57*, *64*, *70*). Along with male-mediated female fecundity and larval viability as other contributors to our male scores, such male traits have the potential of being inherited by the offspring and so confer some indirect fitness benefits to females. We cannot exclude the possibility of some differential male allocation in response to female variation (e.g., courtship vigor), but we expect such differences to mediate female biases rather than overriding them, given that female acceptance (and so likely some mating decision) is necessary for copulation to occur in *D. melanogaster* (*55*–*57*). Combined with the lack of differential sperm allocation as one form of male mate choice (*71*), the effect of female mate choice here was likely stronger than that of male choice, at least during these direct mate interactions. If so, our findings are in line with theoretical and empirical studies indicating positive correlations between mate choice and fitness-related traits (*72*–*74*). By mating with high-quality males, females may gain indirect benefits (*43*–*46*) [but see (*47*, *48*)], even if these do not always offset the costs of mating itself (*52*).

Nonadditive effects also contributed to MS, contingent on the female reproductive value. These results extend prior findings in other taxa that male quality and compatibility can jointly influence mate choice (*75*, *76*), allowing females to balance the relative fitness benefits gained from either source of mate variation. To the extent that mating decisions are condition or context dependent, differences in optimal mate choice between females can contribute to the maintenance of variability in both compatibility genes and male sexually selected traits, even if the latter are under strong directional selection (*75*). Further variation can be explained by the fact that genetic quality can only be advertised via phenotypic traits that are themselves also influenced by environmental factors, which ultimately weakens the correlation between these traits and the underlying genetic quality (*18*, *19*). Yet, the advertisement of intrinsic quality by males and its cognitive assessment by females are more likely to evolve than female preference for compatible genes (*77*). The latter is thought to be limited to specific genetic systems (e.g., major histocompatibility complex) that may be communicated via pheromones (*75*, *76*). However, while such mate choice generally appears to be weak at best (*78*, *79*), systems that are based on polygenic male × female interactions should be even more evolutionarily constrained (*77*). What precise mechanisms could explain the mating outcomes in our experiment may be resolved in future studies.

Besides chemical communication, the potential for nonadditive effects (or putative effects of genetic compatibility) is likely greater in postmating processes due to numerous physical and molecular interactions between ejaculates and the female’s reproductive tract or ova (*11*, *80*). One of the most direct mechanisms of cryptic female choice is the ejection of sperm after mating to bias sperm storage toward favored males (*28*, *81*, *82*). Here, we found females to delay sperm ejection when the second male was either of higher quality or more compatible with the female, thereby giving their sperm more time to enter storage organs and displacing resident sperm (*58*). Because the relative importance of male quality and compatibility shifted from the former to the latter with the increasing number of resident sperm, it is possible that a sperm-female interaction becomes more important with intensifying competition among sperm (i.e., more first-male sperm defending the storage site). This pattern extends prior findings in *D. melanogaster*, which reported a genotypic male-female interaction underlying female sperm ejection and a phenotypic interaction between relative sperm numbers and ejection time in determining the relative sperm representation in storage (*35*). Our results further indicate that females may not only be able to discriminate and bias among rival sperm but also to do so based on both additive and nonadditive effects, or that factors in the ejaculate mediate female sperm ejection, storage, or utilization in such a manner. Such female discrimination or male triggers would likely be mediated by molecular interactions, given the numerous functional proteins in both the ejaculate and female secretions, often along with specific receptors (*26*, *27*). The relative abundance of ejaculate proteins varies considerably between genotypes in the same *D. melanogaster* population as in the current study (*83*), thus indicating the potential for differential male triggers or molecular interactions with given females. Identifying the molecular basis of these interactions would be an exciting, albeit challenging, avenue toward a deeper understanding of the processes underlying cryptic female choice or potentially postmating sexual conflict (*84*).

The relative sperm representation in storage and the relative paternity shares as the ultimate fitness outcome were influenced by interactions between the female reproductive value and either male-female compatibility or male quality, respectively. It thus appears that MS and paternity as the initial and final events of the reproductive process, respectively, were both primarily influenced by male quality [here for logistic reasons not separating differential offspring viability from competitive fertilization success between males sensu (*85*)]. Yet, male-female compatibility also contributed to these processes, most prominently during the sperm displacement phase that determines the relative sperm representation in storage by the time of female sperm ejection (*35*, *61*). Given that even the highest-quality male is not necessarily the most optimal mate for all females, such nontransitive effects in the reproductive process might facilitate selection against incompatible haplotypes or promote genetic diversity in the offspring (*8*–*12*).

Our findings of stage-dependent reproductive biases, with varying contributions of additive and nonadditive effects, have implications for the broader theoretical and conceptual framework of mate choice and sexual selection. These patterns could indicate a trade-off for females between choosing the highest-quality male and mating with multiple males to also ensure compatibility. Theoretical models predict that compatibility-based selection can emerge even when males vary in their genetic quality, but only when incompatibility is very costly and postmating processes mediate selection for compatible offspring (*86*). Females may then relax their preference for high-quality males and mate with a wider range of males, thereby reducing directional selection on male indicator traits (*86*). However, if many high-quality males are available in a population, females can benefit from both male quality and compatibility by mating with several of these males, now ensuring compatibility without reducing selection for good genes (*86*). This general theoretical framework may also apply to populations of *D. melanogaster*, thus explaining the complex interactions that ultimately favored the paternity of higher-quality males in a context-dependent manner. Future experiments might be able to expand our results and examine the effects of the different selective processes on evolutionary change at the population level, particularly under varying environmental conditions that could affect male quality and its importance relative to genetic compatibility. Furthermore, if postmating processes are the main source of biases for higher genetic compatibility, it would also be interesting to explore how the benefits of increased compatibility outweigh the costs of mating with multiple males to achieve it.

Disentangling the different processes leading to paternity biases in response to male attributes moves our understanding an important step closer to a possible functional explanation for complex three-way interactions between females and rival males (*35*). Beyond these interactions, the relative importance of the different processes and episodes of selection explored here also inform on how mate choice and competition contribute to reproductive success, which is ultimately important to understand the role of sexual selection in population adaptation (*87*) and divergence (*88*). As also shown here, it is often challenging to unambiguously differentiate female- from male-driven contributions, because biasing fitness between competitors requires some level of competition in the first place [particularly in postmating processes (*34*)]. Yet, variation in female biases (or relative sperm competitiveness within a given female reproductive tract) harbors the potential to contribute to the maintenance of phenotypic and genotypic variation in natural populations. Such differential biases could counteract the gradual depletion of genetic variation that would be expected under directional sexual selection resulting from a continuous reproductive advantage of higher-quality males (*7*, *13*, *14*). Here, we showed that some of these differential biases could derive from varying reproductive values among females themselves. What remains to be established, however, is how the contributions to maintaining genetic variation compare between genetic compatibility (*8*–*12*) and other processes such as condition-dependent variation in male pre- and postmating fitness (*89*, *90*).

## MATERIALS AND METHODS

### Fly stocks

We performed all our experiments with *D. melanogaster* (wild-type strain LH_m_) that were genetically transformed so that sperm heads express either GFP or RFP (*61*). These fluorescent labels facilitated counts of and discrimination between sperm from different males. We used isogenic lines that had been generated by 15 generations of full-sibling inbreeding leading to a theoretical level of homozygosity of 96% (*91*). To overcome potential inbreeding effects, we generated heterozygous but still near-clonal lines (henceforth “genotypes”) by crossing males of one isogenic line and virgin females from another (all genotypes were fully independent, that is, generated with different isogenic lines). These heterozygous lines were shown to exhibit heritable reproductive traits and repeatable estimates of behavioral and life-history traits (*58*, *62*, *92*). Where sex-specific assays involved standard females or competitor males, these represented pairwise crosses between isolines that were independent of any focal lines or between the sexes of standard genotypes. We maintained all flies in a climate chamber at constant 24°C, 60% humidity, and a 14:10 light:dark photoperiod. All flies used in our experiment were 3- to 4-day-old virgin individuals.

### Estimating genetic quality and compatibility

To estimate the intrinsic quality of each male genotype and the compatibility of each male-female combination of genotypes, we measured a suite of fitness traits under noncompetitive and competitive conditions. We crossed males of 10 GFP and 10 RFP genotypes with females of 10 separate RFP genotypes (Fig. 1B). This resulted in 200 genotypic crosses, each replicated across 15 pairs (i.e., 3000 pairs in total).

#### Noncompetitive fitness assays

For each replicate, we recorded the time between introducing the pair to the vial and the start of copulation (i.e., “mating latency”), before discarding the male and allowing the female to lay eggs for 3 days. Here, we quantified the egg-to-adult viability of the eggs laid on the second day and the total number of adult offspring eclosing across all 3 days and determined the correlations between them using repeated-measures correlations (fig. S3) (*65*). Table S8 presents the partitioned variance between the additive and nonadditive effects of male and female genotypes for all traits.

### Competitive fitness assays

We measured the competitive ability of all male genotypes in two different group-mating experiments. First, we assessed the relative MS among all pairwise male-male genotypic combinations (separately for GFP and RFP genotypes) by competing two males of different genotypes with a standard female (single genotype, separate from all male genotypes). To distinguish competitor males, we marked one of each pair of males with a paint dot on the thorax, balanced across trials to avoid biases among genotypes due to marking. In total, we had 90 genotypic combinations (45 GFP and 45 RFP) across two marking treatments and five replicates per combination (i.e., *N* = 900 trials). To estimate MS, we first introduced the two virgin males to the vial and then added the virgin standard female and recorded which male was the first to mate. Based on the mean number of matings achieved per genotypic combination, we calculated genotype-specific David’s scores (*66*, *67*) as implemented in the R package compete (*93*) to rank the genotypes based on MS.

Second, we estimated the “global fitness” of each male genotype (competitive paternity), which combined competitive mating and fertilization success, as well as larval competition. To this end, we combined in each vial one focal male (GFP or RFP genotype), two competitor males of the opposite color and three RFP females. Females and competitor males within a vial were each derived from their own, unrelated isoline cross. We then tested each focal genotype in two different genetic compositions of females and competitor males, each replicated four times (i.e., total of eight vials per focal genotype). We allowed the six individuals per vial to interact freely for 1 week (transferred to a fresh vial every 2 to 3 days), then discarded the adult flies, reared all progeny, and quantified global fitness as the proportion of focal-male progeny based on ubiquitous GFP expression in the offspring of the GFP males (either focal or competitor) under an Olympus SZX12 fluorescence stereomicroscope (Olympus Schweiz AG, Wallisellen, Switzerland). We omitted the first vial of each replicate as the first mating of each female (and thus progeny over 1 to 2 days) could be more random and disproportionately contribute to total fitness due to noncompetitive fertilization until remating.

### Indices of genetic compatibility and male quality

As an overall estimate of genetic compatibility (i.e., male-female fitness, *w*_ij_), we averaged for each male-female genotypic combination the mating latency (*L*: additive inverse, so higher values indicate higher fitness), egg-to-adult viability (*V*), and total progeny production (*P*), respectively. To equalize contributions between the three variables, we range-standardized each across all genotypic combinations {i.e., $z{\bar{L}}_{ij}=[{\bar{L}}_{ij}-\min(\bar{L})]/[\max(\bar{L})-\min(\bar{L})]\}$ so that all values ranged between 0 and 1. Then, we averaged the three standardized trait means for each combination (higher scores indicating higher compatibility), following Eq. 1

$$w_{ij}=(z{\bar{L}}_{ij}+z{\bar{V}}_{ij}+z{\bar{P}}_{ij})/3$$ (1)

where *i* is the male genotype, *j* is the female genotype, and the bar indicates the mean and *z* the standardization of the variables. To validate these compatibility scores, we generated 1000 datasets of equal size for each variable separately, using stratified resampling with replacement within male-female combinations, and then calculated *w_ij_* for all genotypic combinations within each dataset.

To estimate the genotype-specific male fitness (*w_i_*) as an index of genetic quality (i.e., reproductive value), we calculated for each male genotype the grand means across all 10 female genotypes (i.e., *w*_*i*,1–10_) of the same three noncompetitive fitness variables as above. In addition, we estimated the genotype-specific MS based on David’s scores and PG as global fitness in group vials. After range-standardizing all five variables as above, we calculated *w_i_* as described in Eq. 2

$$w_{i}=(z{\bar{L}}_{i}+z{\bar{V}}_{i}+z{\bar{P}}_{i}+z{\bar{\mathrm{MS}}}_{i}+z{\bar{\mathrm{PG}}}_{i})/5$$ (2)

where *i* is the male genotype, the bar indicates the grand mean, and *z* means the standardization of the variables. Particularly because the competitive assays were based on separate sets of flies per genotype and the David’s scores were necessarily limited to a single value per male genotype (i.e., preventing error estimation), we again resampled 1000 datasets for each variable as above. For David’s scores, we randomly resampled with replacement the competitive outcomes for each pair of male genotypes and then calculated the corresponding dominance scores for each of the 1000 datasets. For global fitness, we resampled the paternity shares of replicate vials of each genotype. Combining the datasets for each of the five fitness traits, we calculated *w_i_* within each of the 1000 iterations and then the genotype-specific means with 95% confidence interval across all iterations (Fig. 1C). Last, analogous to the males, we also estimated female genotypic quality as the mean of *V* and *P*: $w_{j}=z{\bar{V}}_{j}+z{\bar{P}}_{j}$. Figure S7 shows that the variation in the difference in genotypic quality and male-female compatibility was not biased in one direction or the other.

### Genetic quality versus genetic compatibility

To test whether pre- or postmating male-male competition or female choice favors a male of higher intrinsic quality or of higher compatibility with the female, we staged either simultaneous or sequential competition between GFP and RFP male genotypes. To this end, we ranked both GFP and RFP males by their *w_i_* values. For each color, we then selected the top three and bottom three genotypes to represent high- and low-quality genotypes, respectively. We assigned pairs of male genotypes (one GFP and one RFP) each to a pair of female genotypes. In half of these combinations, the two male genotypes contrasted primarily in *w*_i_ (i.e., low versus high quality) but exhibited comparable compatibility scores with both females. In the other half of these combinations, the two male genotypes were derived from the same quality category (either high or low) but contrasted in *w_ij_* between the two females (i.e., one male more compatible with the first female and the other male more compatible with the second; Fig. 1B). We replicated each of these combinations 10 times, five times in either mating order where females were mated to the two males sequentially.

#### Competitive mating

To evaluate the relative effects of male quality and male-female compatibility on premating male-male competition or female choice (MS), we combined two rival males and one virgin female in a vial in each of the assigned genotype combinations to vary relative quality and compatibility (see above). Upon the start of copulation, we removed the unmated male and determined which rival male mated based on the presence or absence of the ubiquitous GFP expression of the unmated male. Each trio of genotypes was replicated 10 times (24 trios × 10 replicates = 240 trials).

#### Competitive fertilization success

To estimate the proportion of all progeny that were sired by the second male (P_2_), we sequentially mated two males to a female, in 10 replicates for each of the two possible mating orders among competitors (36 combinations × 2 mating orders × 5 replicates = 360 trials). We selected genotypes that varied in the magnitudes of difference in quality and compatibility (♂_2_ − ♂_1_) between competing males. We then mated 3-day-old virgin females to the first male (RFP or GFP) and, 2 days later, to the second male (opposite color). Refractory females were given three more remating opportunities as necessary on the third to fifth day. After the second mating, we discarded the male and allowed the female to lay eggs for 3 days in a fresh vial. We then reared the offspring and calculated the proportion of all progeny that were sired by the second male (P_2_), with paternity based on the ubiquitous GFP expression in the offspring of GFP males under a Leica MS5 fluorescence stereomicroscope (Leica Microsystems, Heerbrugg, Switzerland).

#### Postmating processes

To assess the ejection latency (time from the end of copulation with the second male to sperm ejection by the female) and proportion of the sperm remaining in the storage organs that were from the second male (S_2_), we repeated the competitive fertilization experiment, but this time focusing on the reproductive processes after mating instead of paternity. Immediately after the second mating, we transferred each female to a well of a 24-well culture plate, covered with a glass coverslip (secured with drops of rubber cement in two corners). For up to 5 hours after mating, we checked (under a stereoscope) each well every 5 to 10 min for the appearance of the sperm mass that is routinely ejected by the female, containing both displaced first-male and excess second-male sperm (*45*, *48*). We recorded the time from the end of the copulation to female sperm ejection, which contributes to reproductive outcomes through variation in the time window during which the last-male sperm can enter the storage organs and displace resident sperm from them (*58*, *68*, *82*). Subsequently, we froze both the ejected mass and the female for later sperm counts under an Olympus BX51 fluorescence microscope. On the basis of these sperm counts, we quantified the total number of sperm transferred by the second male, the first-male sperm still residing in the female reproductive tract at remating, the proportion of the first male’s sperm that were displaced and ejected and the proportion of second-male sperm among all sperm stored after ejection (S_2_). S_2_ has repeatedly been shown to be positively correlated with paternity (P_2_) in *D. melanogaster* (*58*, *61*, *62*, *69*). To test for potential fitness consequences of mating with higher-quality or more compatible males, we calculated the proportion of each female’s total offspring number that were produced after remating (Pr). Specifically, we calculated Pr as the number of progeny that a female produced after remating divided by the total number of progeny she produced from the first mating to 3 days after the second mating. We statistically accounted for the variation in the first oviposition period by including the remating interval as a covariate, whereas the period after remating was always 3 days. In this context, Pr essentially captures how remating with the second male changes the female’s rate of progeny production relative to that before remating (i.e., first male only).

### Statistical analyses

We carried out all statistical analyses in R 3.6.2 (*94*), using the lme4 package (*95*) to compute all mixed-effects models and with all continuous predictor variables standardized to ~*N*(0,1). We conducted generalized mixed-effects logistic regressions for all binary or proportional response variables (i.e., MS, S_2_, P_2_, and Pr) and a linear mixed-effects model for ejection latency (log-transformed). We started with a set of biologically meaningful models including all relevant covariates [depending on a priori predictions based on the literature; (*62*, *68*, *69*, *96*)] and their two- and three-way interactions. We tested all models for the effect of the difference in quality and the difference in male-female compatibility (♂_2_ − ♂_1_), and their interaction. Premating MS included only the difference in quality and the difference in compatibility. The postmating traits (ejection latency, S_2_, P_2_, and Pr) additionally included traits such as the number of the first-male sperm still in storage at remating, number of the second-male sperm transferred, number of progeny produced before remating, and/or remating interval. All models included random effects for male 1, male 2, female genotypes, and male 1–male 2 combination, and generalized models had an additional observation-level random effect where needed to account for overdispersion. Although these genotypes were not strictly random but rather selected based on their attributes, fixed-effects models were impractical given the necessary model structure. We tested models for collinearity using the check_collinearity function from the performance package (*97*). We reduced complex models by stepwise model simplification (with a cutoff of *P* > 0.10) based on likelihood ratio tests, using the lrtest function of the lmtest package (*98*). However, for completeness, we retained focal main effects (e.g., male quality and male-female compatibility) even if they were not statistically significant. Sample sizes varied between analyses because of missing data in focal traits (e.g., no remating, no sperm ejection, or no offspring between matings combined with no first-male offspring after remating, thus suggesting no successful first mating).
