# Supplementary material for: Interplay between male quality and male-female compatibility across episodes of sexual selection
Source: Sci Adv. 2023 Sep 29;9(39):eadf5559. doi: 10.1126/sciadv.adf5559 (PMC10541500; doi:10.1126/sciadv.adf5559)
Supplement: Supplementary file 1 — Figs. S1 to S7 Tables S1 to S8 [file sciadv.adf5559_sm.pdf]

Science Advances
AAAS

Supplementary Materials for

Interplay between male quality and male-female compatibility across episodes of sexual selection

Hayat Mahdjoub *et al.*

Corresponding author: Rassim Khelifa, rassim.khelifa@concordia.ca; Stefan Lüpold, stefan.luepold@ieu.uzh.ch

Sci. Adv. **9**, eadf5559 (2023)
DOI: 10.1126/sciadv.adf5559

This PDF file includes:

Figs. S1 to S7
Tables S1 to S8

Supplementary Figures

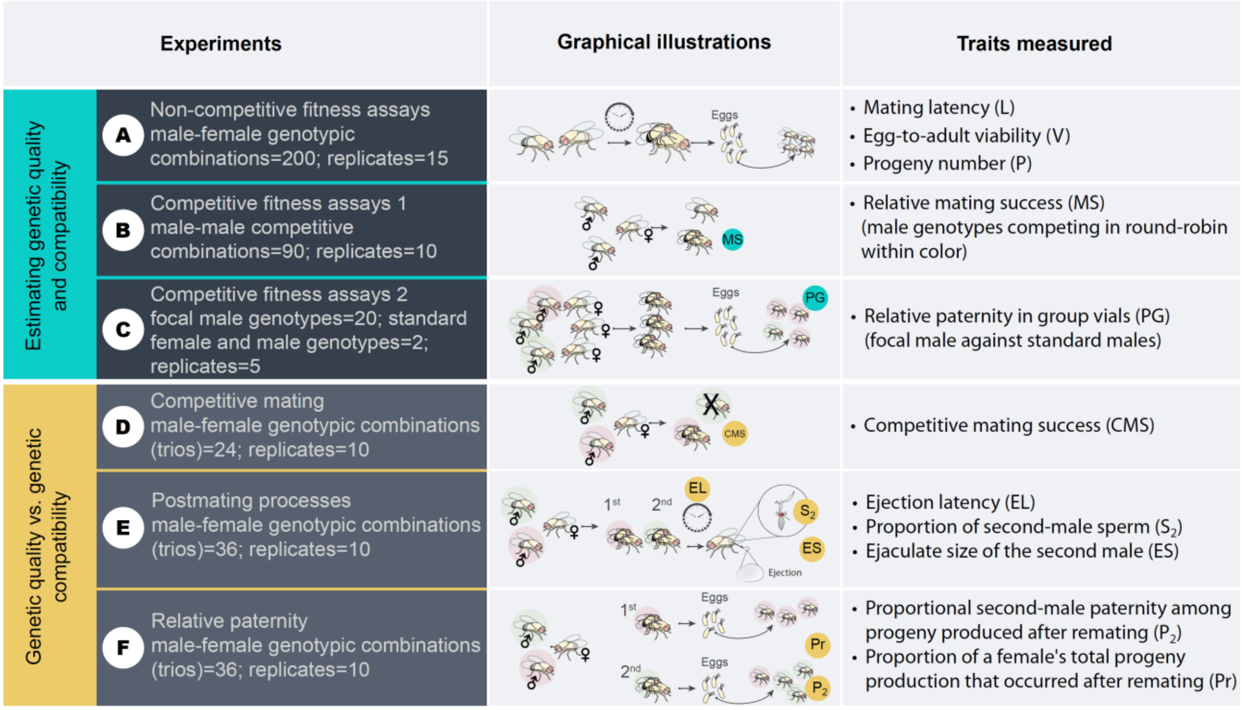

Fig. S1. Overview of all assays, their experimental designs, and traits measured.

The top half (A−C) summarizes the initial assays that were used to quantify male-female compatibility and male quality, using both non-competitive and competitive designs. The bottom half (D−F) describes the final experiments competing males of varying compatibility or quality scores against one another to determine the relevant contributions of the two factors.

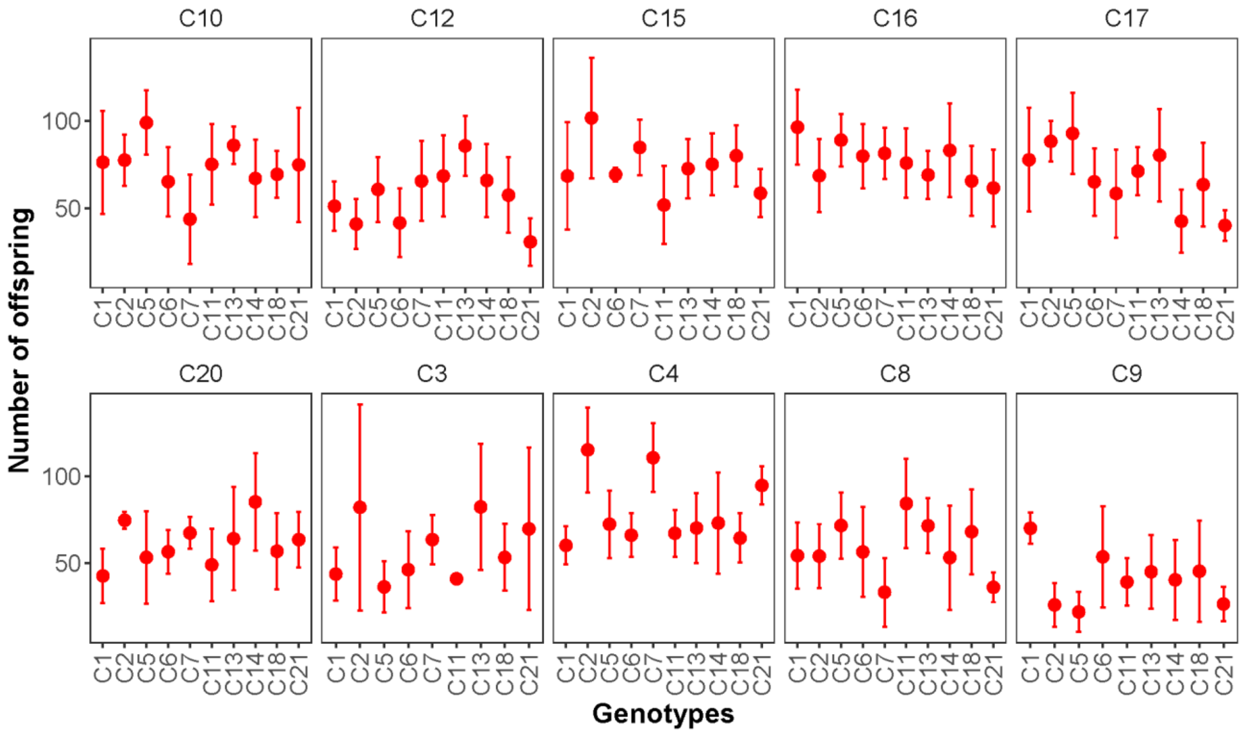

Fig. S2. Productivity of each male-female combination using RFP males, contributing to compatibility scores.

Total number of offspring for each male-female combination of the 10 RFP genotypes (x-axes) with each of the 10 RFP female genotypes (panels). Error bars are 95% confidence intervals.

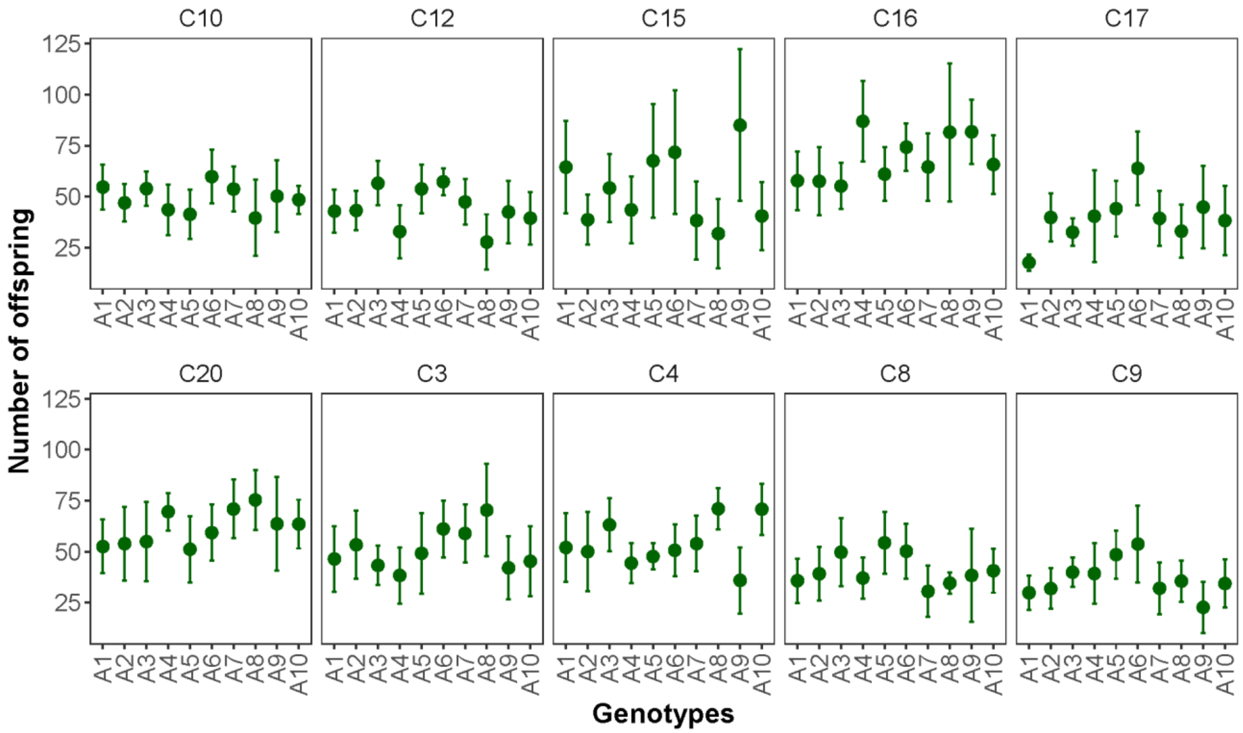

Fig. S3. Productivity of each male-female combination using GFP males, contributing to compatibility scores.

Total number of offspring for each male-female combination of the 10 GFP genotypes (x-axes) with each of the 10 RFP female genotypes (panels). Error bars are 95% confidence intervals.

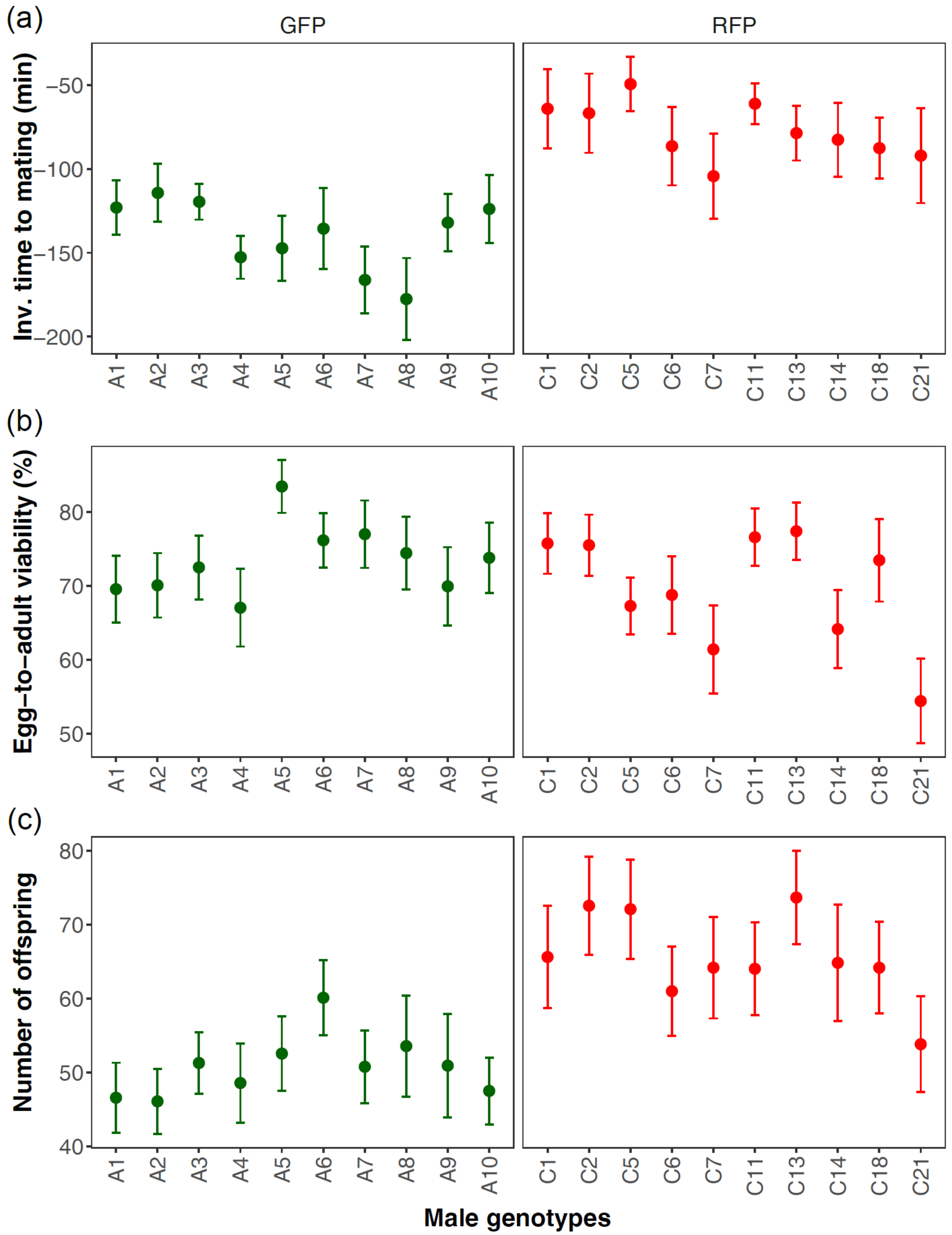

Fig. S4. Non-competitive traits contributing to genotype-specific quality scores.
Copulation latency [additive inverse, in minutes] (a), egg viability (b), and the number of progeny (c) for each of the 10 focal RFP and GFP genotypes, across the 10 female genotypes that each male genotype mated with. Values reflect the genotype-specific grand means with 95% confidence intervals, based on 1,000 bootstrapped iterations (stratified by genotypic combinations).

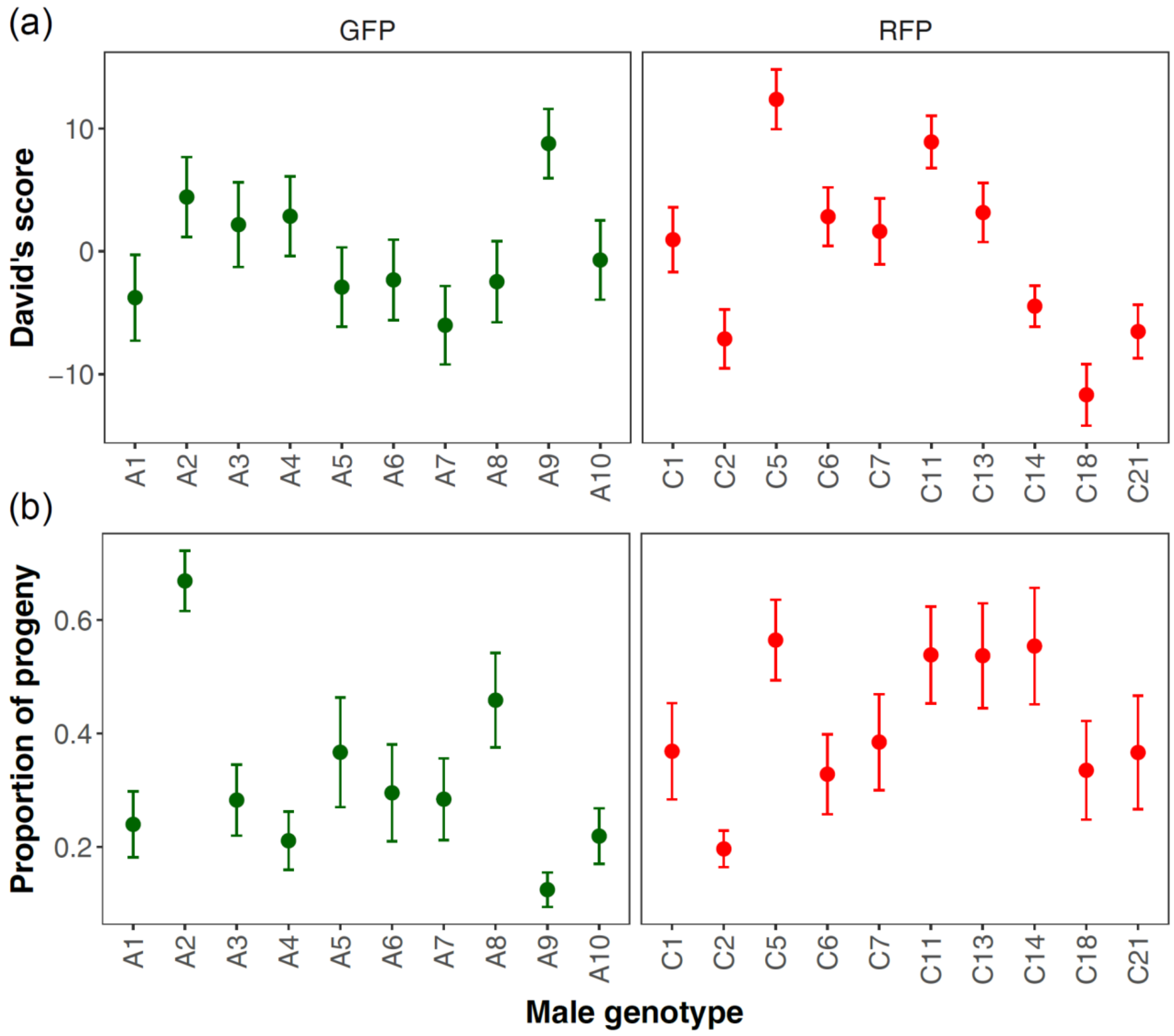

Fig. S5. Competitive traits contributing to genotype-specific quality scores.

Relative mating success as expressed by David's scores (a) and the proportion of progeny in competitive mating trials against two rival males (b) for each of the 10 focal RFP and GFP genotypes in *Drosophila melanogaster*. The David's score is a dominance ranking index, here based on competitive trials of each focal genotype against all 9 rival genotypes, with dyadic winners being those males that mated with the standard female. Since the hierarchy was based on the number of successes across the 10 replicate trials per genotypic combination (i.e., resulting in single values), we calculated 95% confidence intervals across the 9 combinations per genotype by bootstrapping outcomes within genotypic combinations ($N = 1{,}000$ iterations). The proportion of progeny (b) was assayed in group vials where a focal male genotype competed with two standard male genotypes for three standard females (expected paternity = 0.33). Values reflect the genotype-specific grand means with 95% confidence intervals, based on 1,000 bootstrapped iterations (stratified by genotypes).

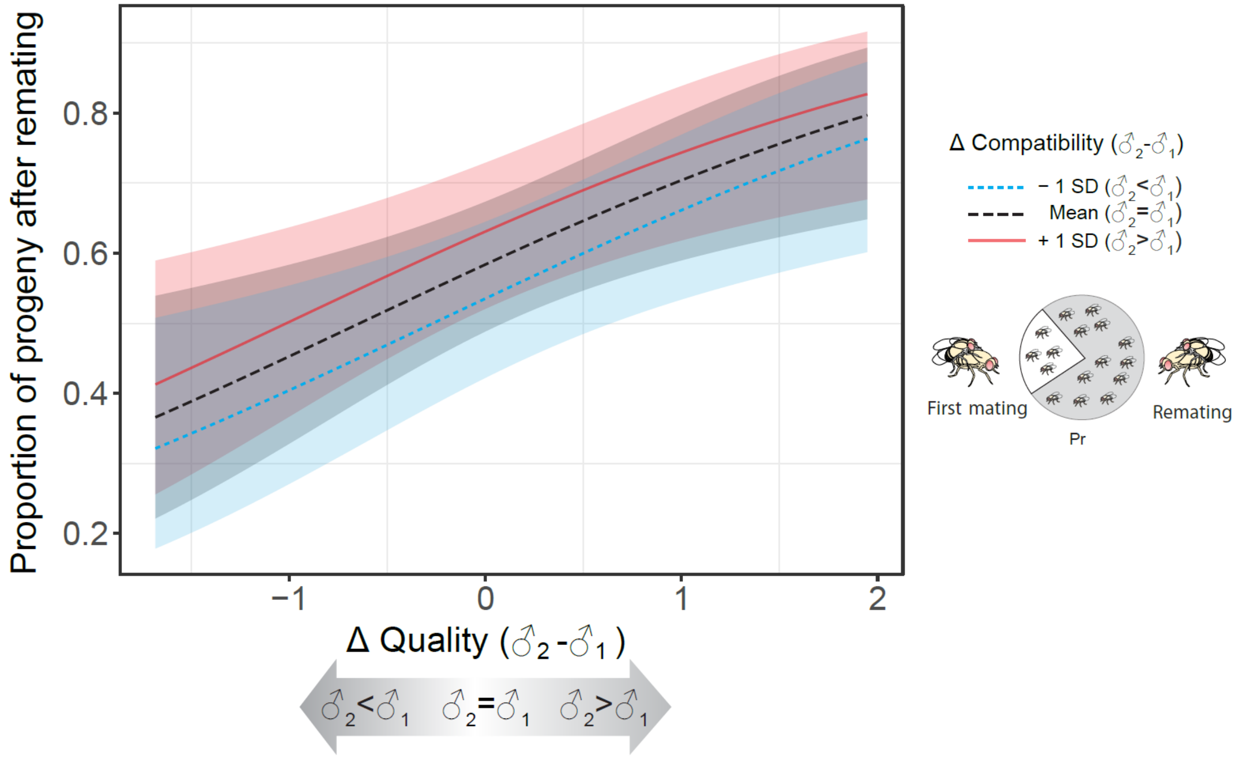

Fig. S6. The effect of the standardized difference (Δ) in male quality and in male-female compatibility on the proportion of all offspring that a given female produced after rather than before remating (Pr) in *D. melanogaster*.

To illustrate the effect of Δ compatibility on the response variable, three levels of Δ compatibility were used (mean, mean-1SD, and mean+1SD). The fitted values were predicted from a generalized linear mixed-effects model.

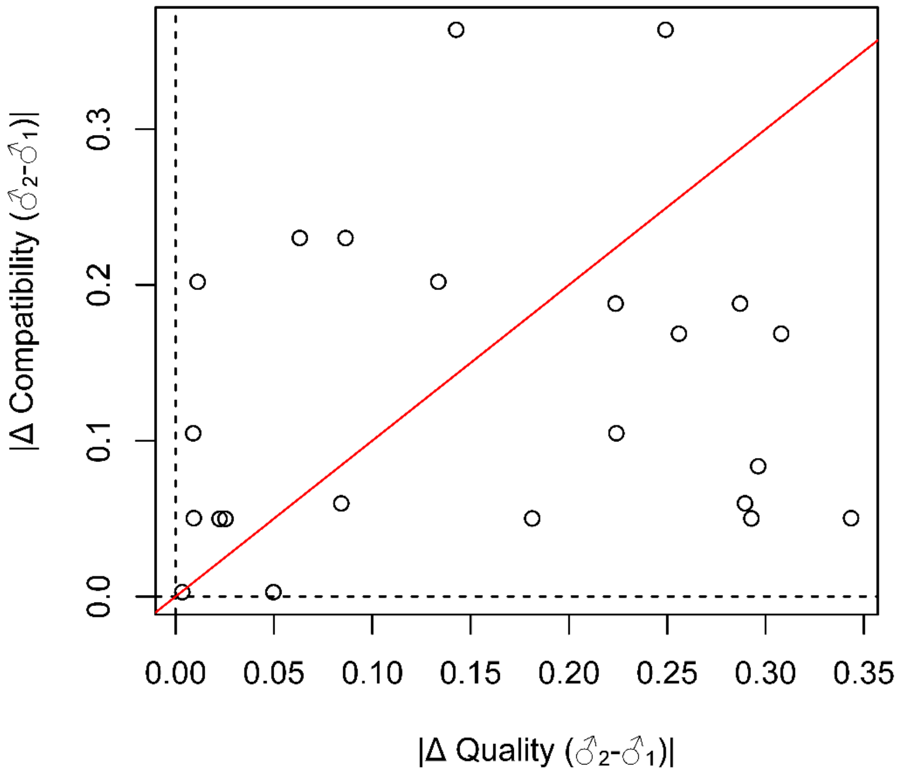

Fig. S7. Comparison between the differences in male quality and compatibility with the female among competing genotypes.

Distribution of the differences in male quality and male-female compatibility of the genotypes used in our competitive experiment. The red line represents equal differences between axes. The dashed lines depict no difference in quality (vertical) or compatibility (horizontal), respectively.

Table S1. Correlations between male quality traits.

All correlation coefficients and 95% confidence intervals were derived from Spearman's rank correlations across the 20 male genotypes in each of 1,000 bootstrapped datasets (see Methods for more detail).

	**Egg-to-adult viability**	**Total number of progeny**	**Relative mating success**	**Relative paternity in group vials**
Mating latency (inverse)	-0.12<br>[-0.40, 0.15]	**0.69**<br>**[0.54, 0.81]**	*0.16*<br>*[-0.02, 0.35]*	**0.30**<br>**[0.01, 0.57]**
Egg-to-adult viability		0.03<br>[-0.29, 0.34]	-0.09<br>[-0.40, 0.22]	0.03<br>[-0.35, 0.38]
Total number of progeny			0.01<br>[-0.24, 0.27]	0.21<br>[-0.11, 0.47]
Relative mating success				0.16<br>[-0.20, 0.49]

Table S2. Effects of male quality and male-female compatibility on mating success.

Summary statistics of a mixed-effects logistic regression of mating success against the differences in quality and compatibility (relative to the corresponding female) between the two males, female quality and the interaction between relative compatibility and female quality. *LLR* = log-likelihood ratio.

Random effects	Variance	Std. Dev.
Male combination	<0.001	<0.001
Female	<0.001	<0.001
Male 2	<0.001	<0.001
Male 1	0.211	0.460

N = 167, Male combination: 12; Female = 8; Male 1 = 7; Male 2 = 8

Fixed effects	Estimate	Std. Error	*LLR*	*P*
ΔQuality	1.177	0.296	13.851	<0.001
ΔCompatibility	-0.214	0.340	0.393	0.531
♀Quality	-0.030	0.208	0.012	0.914
ΔCompatibility×♀Quality	0.518	0.262	3.943	0.047

Table S3. Effects of male quality and male-female compatibility on female sperm ejection. Summary statistics of a linear mixed-effects model assessing the effects of the difference in quality and compatibility values between the two males (♂2-♂1), and ejaculate size on the female sperm-ejection latency (log-transformed) after remating in *Drosophila melanogaster*. *LLR* = log-likelihood ratio.

**Random effects**	**Variance**	**Std. Dev.**
Male combination	0.000	0.000
Male 2	0.000	0.000
Male 1	0.000	0.000
Female	0.000	0.000
Residual	0.312	0.558

N = 227; ♂1-♂2 combination = 34; Male 1 = 16; Male 2 = 16; Female = 9

**Fixed effects**	**Estimate**	**Std. Error**	***LLR***	***P***
ΔQuality	0.089	0.040	4.982	0.026
ΔCompatibility	-0.030	0.043	0.499	0.480
♀Quality	-0.023	0.037	0.390	0.533
Ejaculate size	0.014	0.041	0.126	0.723
ΔQuality×ΔCompatibility	-0.038	0.044	0.747	0.388
ΔQuality×Ejaculate size	-0.021	0.039	0.286	0.593
ΔCompatibility×Ejaculate size	0.060	0.045	1.784	0.182
ΔQuality×ΔCompatibility×Ejaculate size	-0.112	0.041	7.207	0.007

Table S4. Effects of male quality and male-female compatibility on sperm storage. Summary statistics of the generalized mixed-effects model assessing the effect of the difference in quality and compatibility between the two males and the number of resident sperm in the female seminal receptacle on the post-ejection proportional representation of the second male's sperm in the seminal receptacle (S_2) in *Drosophila melanogaster*. *LLR* = log-likelihood ratio.

**Random effects**	**Variance**	**Std.Dev.**
Observation-level random effect	7.475	2.734
Male combination	0.178	0.422
Male2	<0.001	<0.001
Male1	0.003	0.016
Female	<0.001	0.001

N = 241; ♂$_1$-♂$_2$ Combination = 34; Male 1 = 16; Male 2 = 16; Female = 9

**Fixed effects**	**Estimate**	**Std. Error**	***LLR***	***P***
ΔQuality	0.104	0.202	0.205	0.651
ΔCompatibility	-0.167	0.204	0.586	0.444
♀Quality	-0.514	0.206	4.940	0.026
Resident sperm	-1.716	0.216	54.843	<0.001
Ejaculate size	1.352	0.197	42.677	<0.001
ΔQuality×Resident sperm	0.340	0.196	3.229	0.072
ΔCompatibility×♀Quality	-0.492	0.216	5.486	0.019

Table S5. Effects of male quality and male-female compatibility on sperm transfer. Summary statistics of the generalized mixed-effects model assessing the effect of the difference in quality and compatibility between the two males and the number of resident sperm in the female seminal receptacle on the post-ejection proportional representation of the second male's sperm in the seminal receptacle (S_2) in *Drosophila melanogaster*. *LLR* = log-likelihood ratio.

**Random effects**	**Variance**	**Std.Dev.**
Male2	5278	72.65
Female	<0.001	<0.001

N = 219; Male 2 = 16; Female = 9

**Fixed effects**	**Estimate**	**Std. Error**	***LLR***	***P***
(Intercept)	917.36	245.83		
Quality male2	-9.30	222.09	0.064	0.801
Compatibility male2	-141.90	389.47	0.142	0.706
♀Quality	15.06	38.34	0.170	0.681
Male color	135.77	69.66	2.908	0.088

Table S6. Effects of male quality and male-female compatibility on paternity shares. Summary statistics of the generalized mixed-effects model testing for the effects of differences in quality and compatibility between the two males, and female quality on the proportion of the second-male paternity (P_2) in *Drosophila melanogaster*. *LLR* = log-likelihood ratio.

**Random effects**	**Variance**	**Std.Dev.**
Observation-level random effect	4.818	2.195
Male combination	0.410	0.640
Male 2	0.299	0.547
Male 1	<0.001	<0.001
Female	<0.001	<0.001

N = 170; ♂$_1$-♂$_2$ Combination = 34; Male 2 = 16; Male1 = 16; Female = 9

**Fixed effects**	**Estimate**	**Std. Error**	***LLR***	***P***
ΔQuality	0.243	0.242	1.198	0.274
ΔCompatibility	-0.230	0.205	0.908	0.341
♀Quality	-0.281	0.214	1.147	0.284
Remating interval	0.084	0.389	0.021	0.886
ΔQuality×♀Quality	-0.521	0.216	5.194	0.023

Table S7. Effects of male quality and male-female compatibility on offspring production. Summary statistics of the generalized mixed-effects model testing for the effects of differences in quality and compatibility between the two males and remating interval on the proportion of a given female's total offspring produced that both males combined sired after remating as opposed to the offspring produced with the first male only before remating in *Drosophila melanogaster*. *LLR* = log-likelihood ratio.

**Random effects**	**Variance**	**Std.Dev.**
Observation-level random effect	1.737	1.318
Combination	0.096	0.311
Male 2	0.158	0.397
Male 1	<0.001	<0.001
Female	<0.001	<0.001

N = 170, Combinations = 17, Male 1 = 16, Male 2 = 16, Female = 9

**Fixed effects**	**Estimate**	**Std. Error**	***LLR***	***P***
ΔQuality	0.593	0.145	12.675	<0.001
ΔCompatibility	0.132	0.116	1.214	0.271
Remating interval	-0.738	0.226	10.413	0.001
♀Quality	0.212	0.120	3.096	0.078

Table S8. Variance decomposition for different fitness traits.
Variance components of the male, female and female×male random effects for mating latency, total number of progeny, and egg-to-adult offspring viability. *P* values were calculated by likelihood ratio tests between models with and without the female×male interaction, respectively.

	**Random effect**	**vcov**	**sdcor**	**var.comp**	***P***
Mating latency (log-transformed)	Female×Male	0.02	0.15	10.2	0.020
	Male	0.03	0.18	12.0	
	Female	0.00	0.06	3.9	
	Residual	1.18	1.09	73.8	
Number of progeny (log-transformed)	Female×Male	0.01	0.12	6.7	0.587
	Male	0.00	0.00	0.0	
	Female	0.03	0.17	9.9	
	Residual	2.17	1.47	83.4	
Egg-to-adult viability (arcsine-transformed)	Female×Male	0.01	0.10	14.1	0.028
	Male	0.00	0.07	9.1	
	Female	0.01	0.11	15.7	
	Residual	0.20	0.44	61.0	

var.comp (variance component) here is the percentage of variance explained by the random effect.
